# Transforming Lung Cancer Management: A Promising Case Study of Immune Checkpoint Inhibitor Success Following a Multidisciplinary Approach

**DOI:** 10.3390/diagnostics14192159

**Published:** 2024-09-28

**Authors:** Tadashi Nishimura, Hajime Fujimoto, Takumi Fujiwara, Tomohito Okano, Taro Yasuma, Esteban C. Gabazza, Hidenori Ibata, Tetsu Kobayashi

**Affiliations:** 1 Department of Pulmonary Medicine, Mie Chuo Medical Center, Hisaimyojin-cho 2158-5, Tsu 514-1101, Mie, Japanibatah@drive.ocn.ne.jp (H.I.); 2 Department of Pulmonary and Critical Care Medicine, Mie University Faculty and Graduate School of Medicine, Edobashi 2-174, Tsu 514-8507, Mie, Japan; 3 Department of Genomic Medicine, Mie University Hospital, Edobashi 2-174, Tsu 514-8507, Mie, Japan; 4 Department of Immunology, Mie University Faculty and Graduate School of Medicine, Edobashi 2-174, Tsu 514-8507, Mie, Japan

**Keywords:** lung cancer, atezolizumab, immune checkpoint inhibitor

## Abstract

A 54-year-old female patient diagnosed with Stage IIIb squamous cell carcinoma (cT2aN3M0) initially received chemoradiotherapy. Two years after initial treatment, cancer relapse led to the administration of nivolumab, which was halted due to the development of drug-induced pneumonitis. Subsequent management with prednisolone and eight different cytotoxic agents failed to prevent metastasis to the cervical lymph nodes. The tumor’s programmed death-ligand 1 (PD-L1) expression rate was recorded at 10%. Four years after her diagnosis, the patient received a ninth-line therapy combining cisplatin, gemcitabine, and necitumumab, followed by palliative neck radiation due to increasing lymph node size. Remarkable tumor regression occurred three months after introducing atezolizumab as the tenth-line treatment, suggesting that previous treatments, particularly radiotherapy and cisplatin, might have enhanced PD-L1 expression, aligning with the existing literature. This case highlights the urgent need for further research to elucidate the intricate interplay between treatment history and PD-L1 expression in squamous cell carcinoma, emphasizing the importance of accumulating case studies to inform therapeutic strategies.

**Figure 1:**
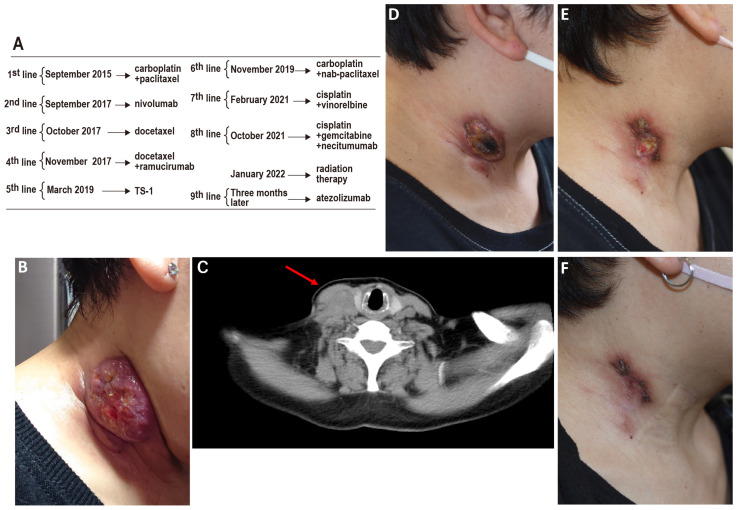
Rapid and marked reduction in right cervical lymph node size post-atezolizumab therapy. Cancer treatment typically adheres to evidence-based protocols established in international clinical guidelines. Despite this, progression can occur even with strict adherence to recommended therapies. We present a challenging case of a 54-year-old female diagnosed with Stage IIIb squamous cell carcinoma (cT2aN3M0), initially managed with chemoradiotherapy ((**A**); 1st-line therapy). Following a relapse two years later, nivolumab was administered ((**A**); 2nd-line therapy). Unfortunately, the emergence of drug-induced pneumonitis necessitated the discontinuation of nivolumab and the initiation of prednisolone therapy. Despite subsequent treatment with eight different cytotoxic anticancer agents ((**A**); 3rd- to 7th-line therapy), metastasis to the cervical lymph nodes continued to progress ((**B**,**C**), red arrow indicates the tumor). Genomic profiling of cervical lymph node samples via next-generation sequencing (FoundationOne^®^, Foundation Medicine, Inc., Cambridge, MA, USA revealed amplifications of fibroblast growth factors (FGF)3, FGF4, FGF19, KIT, platelet-derived growth factor receptor alpha (PDGFRA), Cyclin D1 (CCND1), phosphatidylinositol-4,5-bisphosphate 3-kinase catalytic subunit alpha (PIK3CA), and SRY-box transcription factor 2 (SOX2), along with a tumor mutational burden (TMB) of 11.35 Muts/Mb. Programmed death-ligand 1 (PD-L1) expression, assessed using the 22C3 assay, was found to be 10%. Four years post-diagnosis, the eighth-line therapy (**A**) comprising cisplatin, gemcitabine, and necitumumab was initiated. However, due to the enlargement of the right cervical lymph node, palliative neck radiation therapy consisted of 2 Gy/day for 10 days; in total, 20 Gy was administered (**A**). Radiation was delivered with fractionation over 10 days, rather than 5, to avoid potential bleeding from repeated irradiation due to the overlap in the areas receiving 60 Gy in both 2015 and 2022 [1]. Remarkably, three months later, the administration of atezolizumab as the ninth-line therapy (**A**) resulted in a significant reduction in tumor size (**D**–**F**). Two significant observations emerged from the patient’s treatment trajectory. Initially, while nivolumab induced pneumonitis, atezolizumab did not precipitate this adverse event, suggesting a variance in the side effect profiles of anti-PD-L1 and anti-PD-1 therapies [2]. Secondly, despite modest PD-L1 expression and tumor mutational burden (TMB), the patient experienced long-term efficacy with the immune checkpoint inhibitor. Notably, she had undergone irradiation and cisplatin treatment just before the administration of immune checkpoint inhibitors. The existing literature suggests that both cisplatin and irradiation may enhance PD-L1 expression, potentially explaining the late-line success observed in this case, mirroring other documented instances [3,4]. Growing evidence supports the combination of radiotherapy with immune checkpoint inhibitor therapy [4,5,6], as indicated by similar case reports [7], although others suggest caution [8]. This case exemplifies the unpredictable and individual nature of cancer treatment outcomes and highlights the importance of personalized medicine. It also underlines the potential of revisiting previously failed therapies under altered physiological contexts, such as changes in PD-L1 expression induced by specific treatments. While conclusions drawn from a single case are inherently limited, this patient’s experience offers valuable insights into the complex dynamics between cancer biology, treatment history, and therapeutic response. It underscores the critical need for ongoing research and the accumulation of detailed case studies to enhance our understanding of how treatments can be optimized based on individual patient profiles, providing hope and potentially life-extending options for those facing advanced cancers.

## Data Availability

All data are available upon reasonable request to the first author of this article.
